# *Candida albicans* versus *Candida dubliniensis*: Why Is *C. albicans* More Pathogenic?

**DOI:** 10.1155/2012/205921

**Published:** 2011-09-04

**Authors:** Gary P. Moran, David C. Coleman, Derek J. Sullivan

**Affiliations:** Division of Oral Biosciences, Dublin Dental University Hospital, Trinity College Dublin, Dublin 2, Ireland

## Abstract

*Candida albicans* and *Candida dubliniensis* are highly related pathogenic yeast species. However, *C. albicans* is far more prevalent in human infection and has been shown to be more pathogenic in a wide range of infection models. Comparison of the genomes of the two species has revealed that they are very similar although there are some significant differences, largely due to the expansion of virulence-related gene families (e.g., *ALS* and *SAP*) in *C. albicans*, and increased levels of pseudogenisation in *C. dubliniensis*. Comparative global gene expression analyses have also been used to investigate differences in the ability of the two species to tolerate environmental stress and to produce hyphae, two traits that are likely to play a role in the lower virulence of *C. dubliniensis*. Taken together, these data suggest that *C. dubliniensis* is in the process of undergoing reductive evolution and may have become adapted for growth in a specialized anatomic niche.

## 1. Introduction

Fungi are an important cause of human infection, and yeast species of the genus *Candida* are the most pathogenic fungi. While most *Candida* species are found in the environment, approximately a dozen or so are associated with colonization and infection of humans [1]. *Candida* species are common commensals of the oral cavity, intestinal tract and vagina, with newborns being colonized soon after birth. While these species are innocuous in most individuals, under certain circumstances they can opportunistically overgrow and cause a variety of diseases [2]. These diseases range from superficial infections of the vaginal and oral mucosae, to life-threatening systemic infections that can spread via the bloodstream to organs throughout the body. The risk factors for candidal vaginitis are poorly understood; however, other candidal infections are largely the result of host-related defects. These include depletion of CD4 T cells in HIV-infected individuals, which predisposes to oropharyngeal candidosis, or neutropenia and intestinal surgery, both of which are significant risk factors for systemic infection [1–3]. 

*Candida albicans* is widely recognized as being the most pathogenic yeast species and in the majority of epidemiological studies has been found to be the most common cause of superficial and systemic infections. Other species, such as *Candida glabrata, Candida parapsilosis, *and* Candida tropicalis* have also been associated with most forms of candidiasis and the relative distribution of each species can vary depending on geographic location, patient cohort, and previous exposure to antifungal drugs [2, 4]. In 1995, a new *Candida* species was identified in HIV-infected individuals with oropharyngeal candidosis in Dublin, Ireland [5]. This species, which was subsequently named *Candida dubliniensis*, is very closely related to* C. albicans* with which it shares many phenotypic properties, including the ability to produce hyphae and chlamydospores, traits previously specifically associated only with *C. albicans* [6–8]. Phylogenetic studies indicate that *C. dubliniensis *is the species that is most closely related to* C. albicans*, and it is often quite difficult to discriminate between the two species in clinical samples [9, 10]. Indeed it was only when DNA fingerprinting techniques were applied to the large-scale analysis of *C. albicans* populations in epidemiological studies that the first isolates of *C. dubliniensis* were originally identified [5]. Surprisingly, despite the close phylogenetic relationship of the two species epidemiological data show that *C. albicans* is far more prevalent than *C. dubliniensis*. In particular, in most analyses of systemic infection, *C. albicans* is found in >50% of cases, while if it is identified at all, *C. dubliniensis* has only been found in at most 2-3% of cases [11–13]. This apparent discrepancy between the ability of the two species to cause infection is also reflected in data obtained from comparative studies in a wide range of infection models (e.g., systemic and mucosal) which clearly show that *C. albicans* is significantly more pathogenic than *C. dubliniensis* [10, 14–18].

The identification of virulence-associated factors in *Candida* species is complicated by the fact that they are opportunistic pathogens that usually exist in harmony with the human host as part of the commensal flora and only cause infection when host deficiencies permit. Since it is by far the most pathogenic *Candida* species, *C. albicans* is the best-studied member of the genus in terms of pathogenesis. The most commonly cited *C. albicans* virulence factors include adhesins (e.g., Hwp1 [19] and the Als family [20]), extracellular enzymes (e.g., the secreted aspartyl proteinase (Sap) family [21] and phospholipases [22]), and most importantly of all, the ability to alternate between unicellular yeast and filamentous hyphal forms of growth [23]. Both morphological forms have been shown to be essential for virulence. Hyphae have been proposed to play a major role in adhesion, invasion, and biofilm formation while yeast cells are likely to be important for dissemination and initial colonization of host surfaces [24]. Comparative phenotypic analysis of *C. albicans *and* C. dubliniensis* has suggested that *in vitro* isolates of *C. dubliniensis* exhibit higher levels of proteinase activity, are more adherent to buccal epithelial cells, and undergo phenotypic switching at a higher rate than *C. albicans * [10, 25–27]. In addition, as described earlier, *C. dubliniensis* is the only *Candida* species, other than *C. albicans* that is able to produce hyphae [5, 6]. Given the close relationship between the two species and the fact that they are so alike phenotypically, at first glance, it is difficult to understand why there is such disparity in the capacity of *C. albicans* and *C. dubliniensis* to colonise and cause disease in humans. This short review appraises recent findings that help to clarify this conundrum and explain how *C. albicans* appears to have evolved to be a better commensal and opportunistic pathogen than *C. dubliniensis*.

## 2. Comparative Genomic Analysis of *C. albicans* and * C. dubliniensis*

The *C. albicans* genome sequence was first published in 2004 [28], with improved annotation and analysis subsequently reported in 2005 [29] and 2007 [30]. In an early attempt to identify genomic differences that might serve to explain the disparity in the virulence of *C. albicans *and* C. dubliniensis*, Moran et al. cohybridized genomic DNA from each species to *C. albicans* whole genome microarrays to identify genes that are only present in *C. albicans *[31]. This relatively crude experiment suggested that there are 247 (approx. 4%) *C. albicans* genes that are either absent or highly (i.e., >60%) divergent in the *C. dubliniensis* genome. Interestingly, several genes strongly associated with *C. albicans* virulence are included in the list of absent/divergent genes. In 2009, in order to further investigate the genetic differences between the two species the Wellcome Trust Sanger Institute sequenced the entire *C. dubliniensis* genome [32]. Comparison of the two genome sequences revealed that, despite major karyotypic differences, the genomes of the two species are remarkably similar with 96.3% of genes exhibiting >80% identity, while 98% of genes are syntenic, thus, confirming the very close phylogenetic relationship and the relatively recent divergence of the two species (estimated to have occurred approx. 20 million years ago [33]). When transposable elements were discounted, comparison of the two genome sequences revealed that there are 29 *C. dubliniensis*-specific genes and 168 *C. albicans*-specific genes. The majority of the differences observed between the two species can be accounted for by the expansion of gene families in *C. albicans*, many of which have been previously associated with virulence. In particular, genes missing from the *C. dubliniensis* genome include those encoding hypha-specific virulence factors, such as the cell surface proteins Hyr1 and Als3 and two members of the secreted aspartyl proteinase family (i.e., Sap5 and Sap6), while the gene encoding the well-characterized epithelial adhesin Hwp1 is highly divergent [31, 32]. Hyr1 has been shown to confer resistance to neutrophil killing activity [34] and, along with Hwp1, has been shown recently to play an important role in oral mucosal biofilm formation [35]. Als3 has been shown to play an important role in adhesion to host cells and has been shown to have invasin-like [36] and iron-sequestering [37] activity, while the Saps are well-known virulence factors [21]. The biggest difference in gene family size between the two species is the TeLOmere-associated (TLO) family which is comprised of 14 genes in *C. albicans,* but only two genes in *C. dubliniensis*. Sequence comparisons suggest that the TLO genes encode transcriptional regulators, and preliminary analysis of the phenotype of *C. dubliniensis *Δ*tlo* mutants suggests that these genes may play a role in the control of hypha formation [32]. In addition to these differences, a range of genes appear to be in the process of being lost by *C. dubliniensis*. There are 78 *C. dubliniensis* pseudogenes with intact positional orthologs in *C. albicans*, including genes identified as filamentous growth regulators (FGR) in haploinsufficiency studies [38]. These findings suggest that *C. dubliniensis* is undergoing a process of reductive evolution leading to the loss of genes that have been associated with *C. albicans* virulence. Interestingly, many of these genes are only expressed by the hyphal form of growth and are likely to play a prominent role in host-pathogen interaction.

One of the most prominent phenotypic differences between *C. albicans* and *C. dubliniensis *is their different capacity to tolerate environmental stress, with the former being far more tolerant of thermal, osmotic, and oxidative stress [5, 14, 39, 40]. Indeed, comparative growth at 45°C is commonly used as a simple diagnostic test to discriminate between the two species [41]. Comparative transcriptional profiling analysis revealed that although the two species express similar core stress responses, *C. dubliniensis* mounts a more robust response to thermal stress and a very poor transcriptional response to oxidative and osmotic stress [39]. Forward genetic screens using a *C. albicans* library to try and identify genes that might increase the tolerance of *C. dubliniensis* to environmental stress failed to identify any single gene that could complement oxidative and thermal sensitivity, suggesting that these are likely to be polygenic traits. However, the *C. albicans ENA21* gene, which encodes a sodium efflux pump, was found to increase the salt tolerance of *C. dubliniensis* [39]. Since the *C. dubliniensis* ortholog of this gene appears to be functional but not upregulated in response to the presence of salt, it is likely that the differential salt stress susceptibility of the two species is due to differences in stress-related transcriptional regulatory pathways.

## 3. Comparative Analysis of Hypha Formation by *C. albicans* and * C. dubliniensis*

One of the most important and best-studied virulence factors of *C. albicans* is its ability to switch between yeast and filamentous growth forms (i.e., dimorphism), a trait also shared by *C. dubliniensis *[5]. However, although *C. dubliniensis* is capable of producing germ tubes and true hyphae, it does so far less efficiently than *C. albicans,* both *in vivo* and under a wide range of *in vitro *conditions [16, 42, 43]. Given the perceived importance of dimorphism in *C. albicans* virulence, we have previously suggested that the lower virulence of *C. dubliniensis* may, at least in part, be related to its relatively poor ability to switch between yeast and hyphal forms [16]. Evidence in support of this was obtained from murine systemic infection model studies [14, 15] and the neonatal orogastric infection model [16]. In the latter, stomach and kidney samples in infected animals contained only *C. dubliniensis* yeast cells, while *C. albicans* cells were found in both the yeast and hyphal forms [16]. 

**Figure 1:**
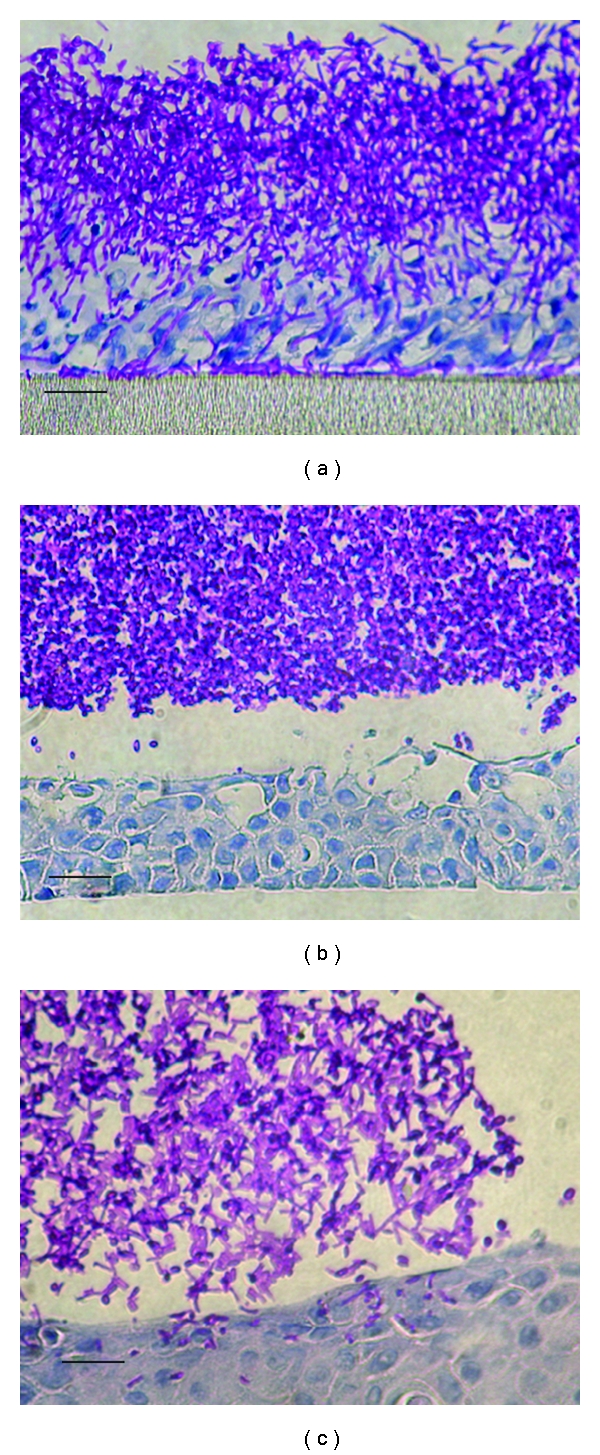
Photomicrograph of *C. albicans* SC5314 and *C. dubliniensis* CD36 infecting oral reconstituted human epithelial (RHE) tissue. (a) *C. albicans* originally grown in nutrient-rich YPD, note the presence of hyphae and extensive tissue invasion and damage; (b) *C. dubliniensis* originally grown in YPD, note the absence of hyphae and the limited level of invasion and tissue damage; (c) *C. dubliniensis* originally grown in Lee's medium, note the increased level of filamentation and invasion. Scale bars, approximately 25 *μ*m.

We have used the RHE model of superficial infection [44] to compare the invasive potential of both species (Figure 1). In particular, in this model, *C. albicans* grows as both yeast and hyphae and invades the tissue causing major damage. In contrast, *C. dubliniensis* grows exclusively in the yeast form in this model, therefore, causing relatively limited tissue invasion and damage [16, 17]. In order to investigate why the two species differ so markedly in virulence in this model and in order to identify novel virulence-associated genes; Spiering et al. compared their global gene expression profiles during the early stages of RHE infection [17]. Both species showed similar expression profiles for ribosomal and general metabolic genes, however, unsurprisingly, *C. albicans* showed increased expression of hypha-specific virulence genes (e.g., *ECE1, HWP1, HYR1, and ALS3*) within 30 minutes of infection. In contrast, *C. dubliniensis* showed a far less robust transcriptional response and no expression of hypha-specific genes. In addition, several genes with unknown function were found to be specifically upregulated in *C. albicans* that are absent from or very divergent in the *C. dubliniensis* genome. One of these genes, named *SFL2* due to its sequence similarity to the transcription factor-encoding gene *SFL1*, encodes a putative DNA-binding heat shock factor protein. When the gene was deleted in *C. albicans*, it resulted in the failure to produce hyphae under a wide range of growth conditions, including the RHE infection model. Interestingly, the Δ*sfl2* mutation had no effect on survival in the murine systemic infection model, although histological analysis revealed that the kidneys of infected mice were infected only with yeast cells, while the kidneys of mice infected with the wild-type parental strains contained both yeast and predominantly hyphal cells [17]. In a subsequent study, it has been shown that the Δ*sfl2* mutant exhibits reduced virulence in a mouse model of gastrointestinal infection, suggesting that Sfl2 is required for the penetration of the gut wall and subsequent dissemination throughout the body [45]. The *C. dublinensis* ortholog of *SFL2* is only 50% identical and is not expressed under the same conditions as the *C. albicans* gene, therefore it is possible that the divergence of this gene and its apparent lack of expression may be partly responsible for its lower virulence.

Recent studies by our group have been directed towards investigating the molecular basis for differences in the signaling pathways responsible for filamentation in the two species. Comparative genome analysis suggests that orthologs of the known components of the major *C. albicans* morphogenetic pathways (e.g., Cph1-mediated MAPK and the Efg1-mediated Ras1-cAMP pathways) are highly conserved in *C. dubliniensis*, so the reduced capacity of *C. dubliniensis* to produce hyphae and express hypha-specific genes such as *SFL2* is unlikely to be due to the absence of regulators involved in these pathways. Forced stimulation of the Ras1-cAMP pathway with a hyperactive *RAS1^G113V^* allele did not result in increased true hypha formation in *C. dubliniensis*, suggesting strong repression of the RAS1-cAMP pathway itself or downstream regulators [43]. One of the most important transcriptional regulators involved in the control of morphogenesis in *C. albicans *is Nrg1, which Staib and Morschhäuser. showed that it is differentially expressed by *C. dubliniensis* when grown on media such as Staib agar [46]. In *C. albicans* this protein targets the negative regulator Tup1 to specific sequences in the promoters of genes involved in hypha formation. *NRG1* expression is rapidly downregulated in *C. albicans* cells incubated under hypha-inducing conditions, including when cells are phagocytosed by murine macrophages, which results in germination and escape from the phagocytes. However, in *C. dubliniensis, NRG1* expression remains high under these conditions, preventing hypha formation and causing cells to remain in the yeast phase, which in the murine macrophage model results in failure to escape from the phagocytes and the death of the fungus [43]. Deletion of the *NRG1* gene in *C. dubliniensis* resulted in an increase in the rate of hypha and particularly pseudohypha formation, which in turn led to increased survival when exposed to murine macrophages as well as increased virulence in the reconstituted human epithelial (RHE) cell model of oral candidosis. Surprisingly, the *C. dubliniensis *Δ*nrg1* mutant was no more virulent than its parent strain in the murine systemic infection model and formed mainly pseudohyphae in infected kidneys, suggesting an additional level of repression preventing true hypha formation *in vivo* [43].

Recent investigations by O'Connor et al. have suggested that repression of filamentation in *C. dubliniensis* is mediated by nutrients. In order to improve our understanding of how environmental signals trigger these pathways O'Connor et al. investigated the effects of nutrient availability on the rate of hypha formation [42]. One of the most common incubation conditions for inducing hypha formation in *C. albicans* is incubation in the nutrient-rich medium YPD supplemented with 10% (vol/vol) fetal calf serum at 37°C. Under these conditions >80% of *C. albicans* cells produced germ tubes/filaments within two hours, in contrast only ~20% of *C. dubliniensis* cells were observed to produce hyphae under the same induction conditions. However, when *C. dubliniensis* cells were incubated in water supplemented with 10% (vol/vol) fetal calf serum (WS) at 37°C, the level of hypha producing cells increased to 90% (i.e., similar to the level of hypha formation by *C. albicans*), suggesting that a nutrient-rich environment, in particular the presence of complex mixtures of peptides, suppressed hypha formation in this species. This was confirmed when the addition of peptone and peptone and glucose was found to significantly reduce the levels of hyphae, suggesting that nutrient starvation is a prerequisite for hypha formation by *C. dubliniensis *[42]. These morphological changes were coupled with changes in the expression of genes encoding key transcriptional regulators, such as *NRG1* and *UME6*, which were significantly altered in WS, with the former downregulated by 70% and the latter upregulated 30-fold. Overexpression of the *UME6* gene (which encodes a protein required for hyphal extension), using a doxycycline-inducible promoter led to *C. dubliniensis* cells being able to produce true hyphae, even in nutrient-rich media such as YPD. Similarly, preculture of *C. dubliniensis* cells in nutrient poor media, such as Lee's medium, pH 4.5, prior to the induction of hyphae in YPDS also resulted in a transient ability of *C. dubliniensis* cells to produce hyphae which increased the ability of these cells to adhere to and invade epithelial tissue in the RHE model (see Figure 1) and increased survival in the murine macrophage infection model [42]. 

These data suggest that factors controlling *UME6* expression in *C. dubliniensis* are repressed by the presence of nutrients, and unlike *C. albicans*, this repression cannot be lifted by a shift to alkaline pH, which occurs when serum is added to the medium. *UME6* is likely to be regulated by Efg1 and Eed1 and is therefore under the control of the Ras1-cAMP pathway. Few studies have investigated how nutrients regulate this pathway in *C. albicans* or *C. dubliniensis*. Preliminary investigations in our laboratory have shown that rapamycin, an inhibitor of the nutrient sensing kinase Tor1 (a kinase that plays a central role in the control of responses to nutrient availability [47]), can stimulate transient hypha formation in *C. dubliniensis* in nutrient-rich YPD serum [48]. This derepression of hypha formation in the presence of nutrients is concomitant with a reduction of *NRG1* and an increase in *UME6* expression. These data suggest that differences in Tor1 activity may play a role in the differential ability of *C. albicans *and* C. dubliniensis* to form hyphae. The molecular basis for the difference in the activity of Tor1 in the two species is currently under investigation.

## 4. Conclusions

Candidal pathogenicity involves the complex interplay of a wide range of virulence-associated factors. Comparative analysis of *C. albicans *and* C. dubliniensis* genomic and transcriptomic data has revealed that the reasons for the differences in the capacity of these two species to cause disease are also complex and are not due to a simple defect in *C. dubliniensis*. Instead these studies have revealed genetic differences in the two species, which, at least in part, may explain the differences in their capacity to tolerate stress and to filament. It is clear that the *C. dubliniensis* genome is missing important virulence genes (e.g., *ALS3* and *HYR1*), is in the process of losing others (e.g., the *FGR* genes), has failed to expand certain gene families (e.g., the *SAP* and *TLO* families), and has undergone some degree of transcriptional rewiring (e.g., the Tor pathway and Sfl2). All of these differences suggest that the main discrepancy between these two closely related species relates to differences in hypha formation and the expression of hypha-specific products (summarized in Table 1). We propose that *C. dubliniensis* is in the process of undergoing reductive evolution, whereby its genetic repertoire is diminishing in comparison with *C. albicans* and their common ancestor. One of the main phenotypic manifestations of this is the narrowing of environmental conditions permissive for hypha formation, perhaps as a result of specialization for survival in a specific (as yet unidentified) anatomic niche where hyphae are not required for colonization or growth. By further investigating the molecular basis for the differences between *C. albicans *and* C. dubliniensis*, we hope to improve our understanding of candidal virulence, in particular the relative contribution of hyphae and hypha-specific proteins to the pathogenesis of candidal infections.

## Figures and Tables

**Table 1:** Comparison of *C. albicans* and *C. dubliniensis*.

	*C. albicans*	*C. dubliniensis*	References
Growth and morphology			
Growth at ≥42°C	Yes	No	[39, 41]
Growth in high salt media	Yes	No	[39, 40]
Hypha formation in YPD + serum	Yes	Poor	[16, 42]
Hypha formation in water + serum	Yes	Yes	[42]
RHE infection model	yeasts and hyphae	yeasts only	[17]

Genome			
Chromosome number	8	9–11 chromosome-sized fragments	[7]
No. of species-specific genes	168	29	[32]
*ALS3 *	Present	Absent	[32]
*HYR1 *	Present	Absent	[32]
*SAP4, 5* and *6 *	All three genes	One gene	[32]
*HWP1 *	Present	Divergent	[32]
*TLO* family	14 genes	2 genes	[32]
